# Biochemical and molecular characterisation of the bacterial endophytes from native sugarcane varieties of Himalayan region

**DOI:** 10.1007/s13205-012-0084-2

**Published:** 2012-09-04

**Authors:** Digar Singh, Anita Sharma, Gurvinder Kaur Saini

**Affiliations:** 1 Department of Biotechnology, Indian Institute of Technology Guwahati, Guwahati, 781039 Assam India; 2 Department of Microbiology, G.B. Pant University of Agriculture and Technology, Pantnagar, 263145 Uttaranchal India

**Keywords:** Endophytes, *Gluconacetobacter*, Isolation, Biochemical, Molecular characterisation

## Abstract

Seven endophytic bacterial isolates were finally recovered from native sugarcane varieties at hilly areas namely Berinag, Champawat and Didihat of Uttarakhand state in northern Himalayan region. New isolates and two standard cultures—*Azospirillum brasilense* and *Gluconacetobacter diazotrophicus*, were evaluated for their morphological, biochemical and molecular characteristics. Morphologically all were rod shaped, Gram-negative bacteria. Their plant growth promotory properties were also assessed which proved isolates RtBn and StBn as IAA producing. Except isolate StBn, all were phosphate solubilising and except RtBn all produced siderophores. Molecular characterisation of the isolates was performed using amplified 16S r-DNA restriction analysis. Similarity index in unweighted pair group method with arithmetic mean programme clustered the isolates according to their geographical distribution. Native isolates showed insignificant similarity with South American strains used as standards. *nifH* amplification was observed with all the isolates used in the study which again establish them as potential N-fixers.

## Introduction

Indian green revolution started in mid 1960s which enabled the food autonomy in country. However, excessive use of inorganic fertilizers and pesticides changed the traditional cultivation practices. The situation has become so alarming that now the role of microorganisms in development of sustainable agriculture is being realised. In order to increase the agricultural production, there has been a tendency to adopt high application rate of fertilizer and irrigation water, often together (Hussain and Jaloud 1995). Crop plants are able to utilise about 50 % of the applied nitrogen fertilizer while 25 % is lost through leaching, volatilisation, denitrification, etc. This causes not only an annual economic loss of three billion US$ but also pollutes the environment (Saravanan et al. 2008). On the other hand, biological nitrogen fixation (BNF) is a microbiological process which converts atmospheric nitrogen into the readily accessible form to plants.

Research to improve crop responses has emphasised the study of nitrogen-fixing bacteria indigenous to rhizosphere, but little is known about the non-rhizospheric nitrogen-fixing bacterial population associated with the plants. These include *Cyanobacteria, Azotobacter, Acetobacter, Azospirillum, Azolla*-*Anabaena* symbiosis, Frankia-non-legume nodulation, *Bacillus, Burkholderia, Pseudomonas, Rhizobium,* etc. (Poblet et al. 2000; Muthukumarasamy et al. 2000; Mendes et al. 2007). Among this group, *Acetobacter diazotrophicus*, a sugarcane endophyte has been reported and studied worldwide (James and Olivares 1998). The presence of *A. diazotrophicus* has also been reported in rice (Flores Encarnacion et al. 1999) and wheat (Kennedy et al. 1997). Nitrogen-fixing microbes inhabit varieties of tissues and organelles in plants such as seeds, roots, stems and leaves (Johri 2006). Plants are benefited extensively by harbouring these endophytic microbes and confer enhanced resistance against pathogens (Compant et al. 2005; Clay and Schardl 2002; Hoflich 2000; Arnold et al. 2003) by introducing antibiotics and siderophores (Ezra et al. 2004; Tortora et al. 2011).

These endophytes from different environmental, geographical and physiological conditions may have resulted in allopatric polymorphism. The present study aims to characterise morphological, biochemical and plant growth promotory (PGP ) properties of the isolates. The ribosomal RNA genes of bacteria, especially those for 16S and 23S-rRNA, are excellent molecular markers for phylogenetic studies because of their functional constancy and their ubiquitous distribution (Amann 1995). Molecular phylogeny of the *diazotrophic* spp. using ARDRA technique subjects the amplified 16S rDNA gene to restriction digestion and thus is termed as the ‘amplified rDNA restriction analyses’. Hence, this work involved the isolation and characterisation of endophytic *Acetobacter* sp. from native sugarcane varieties at high altitudes of Uttarakhand (Himalayan region) and their relatedness with the standard strains.

## Materials and methods

### Collection of plant material for isolation of sugarcane endophytes

Sugarcane samples were collected from hilly areas of Uttarakhand state namely—Berinag, Didihat and Champawat, belonging to different altitudes in the Himalayan region. Endophytes were isolated from the root and shoot portions of the plant samples (Dobereiner et al. 1988). Finally, seven isolates were selected for further studies and assigned the respective codes (Table 1). Two standard bacterial cultures were also used, *Azospirillum brasilense* (MTCC-125) and *Gluconacetobacter diazotrophicus* (MTCC^_^1224).

**Table 1:** Characteristic features of the different isolates

Characteristics	StBn	RtBn	StDd	RtDd	RtChI	RtChII	StCh
Place of isolation	Berinag	Berinag	Didihat	Didihat	Champawat	Champawat	Champawat
Plant portion	Stem	Root	Stem	Root	Root	Root	Stem
Colony morphology							
Configuration	Round	Round	Oval	Oval	Round	Round	Round
Margins	Wavy	Wavy	Smooth	Smooth	Smooth	Smooth	Smooth
Elevation	Convex	Convex	Convex	Convex	Convex	Convex	Convex
Shape	Circular	Circular	Circular	Circular	Circular	Circular	Circular
Pigmentation	White	Creamy	Light creamy	Creamy	Creamy	Creamy	Creamy
Cell morphology							
Gram’s reaction	–	–	–	–	–	–	–
Shape	Long rods	Long rods	Short rods	Short rods	Short rods	Long rods	Long rods
Arrangement	Single	Single	Single	Single	Single	Paired	Single
PGPR properties							
Phosphorous–solubilisation	–	+	+	+	+	+	+
Siderophore–production	+	–	+	+	+	+	+
IAA-production	+(7.2 μg/ml)	+(8.92 μg/ml)	–	–	–	–	–
Biochemical characteristics							
H_2_S production	+	+	+	+	+	+	+
Gelatin hydrolysis	–	–	–	–	–	–	–
Casein hydrolysis	–	–	–	–	–	+	–
Starch hydrolysis	–	–	–	–	–	–	–
Catalase activity	+	+	+	–	–	+	+
Denitrification test	–	–	–	+	+	+	–
Cellulase activity	–	–	–	–	–	–	–

(Positive test ^+^, Negative test ^−^)

### Morphological characterisation of bacterial isolates

Bacterial isolates appeared on semi-solid LGIP medium (K_2_HPO_4_ 0.2 g/l, KH_2_PO_4_ 0.6 g/l, MgSO_4_·7H_2_O 0.2 g/l, CaCl_2_ 0.2 g/l, Na_2_MoO_4_ 0.002 g/l, FeCl_3_ 0.01 g/l, bromothymol blue 0.5 % in 0.2 M KOH, agar 2 %, sucrose 100 g/l at pH 5.5) vials and plates showed typical light or heavy orange-yellow surface pellicle on the medium. Gram staining and morphological studies were also performed on the isolates (Holt et al. 1994).

### Functional characterisation of the bacterial isolates

#### Antibiotic sensitivity test

One millilitre of actively growing bacterial cultures was pour plated in nutrient agar plates. Antibiotic discs (Himedia) of Ceftriaxone (Cf30; 30μg/disc), Clotrimazole (Cl30; 30 μg/disc), Tetracyline (T30; 30 μg/disc), Metronidazole (Mt3; 3 μg/disc) and Amoxyclav (Ac5; 5 μg/disc), were placed at four corners of solidified plates. Plates were incubated for 2–3 days at 30 ± 2 °C. Inhibition zones appeared after the incubation was noted for individual organisms and antibiotic(s).

#### Carbohydrate utilisation studies

Carbohydrate kits (KB009 Hicarbohydrate kit, Himedia) containing 35 different sugars in three sets (A, B and C) were inoculated with bacterial cultures separately and incubated at 30 ± 2 °C for 48 h. After incubation, results were observed and compared according to colour chart of the kit. Carbon source utilisation profiling was used for establishing phylogenetic relationship between isolates and the standard by unweighted pair group method with arithmetic mean (UPGMA), sub programme of online software http://genomes.urv.cat/UPGMA/index.php?entrada=Example2 (Garcia-Vallve et al. 1999).

### Plant growth promotory studies

#### Phosphorous solubilisation

Actively growing bacterial culture(s) were spot inoculated on Pikovaskya medium agar plates with composition; (Yeast extract 0.5 g/l, Dextrose 10 g/l, (NH_4_)_2_SO4 0.5 g/l, Ca_3_(PO_4_)_2_ 5 g/l, KCl 0.2 g/l, MgSO_4_ 0.1 g/l, MnSO_4_ 0.0001 g/l, FeSO_4_ 0.001 g/l, Agar 15 g/l at pH 7.0), and incubated at 30 °C for 3 days. Positive isolates developed transparent zone(s) against white opaque background.

#### Siderophore production (qualitative assay)

Bacterial culture(s) were spot inoculated on chrome-azurol sulphonate (CAS, Sigma-Aldrich, USA) agar plates (Schwyn and Neilands 1987). The medium was poured on sterile Petri dishes; then spot inoculated with 10 μl of each of the bacterial isolate at log phase and incubated for 3–5 days at 30 °C. Positive results were indicated by the formation of an orange-yellow zone around the colonies.

#### Quantification of IAA production

Tubes of YPM (Yeast-Peptone-Mannitol) broth 5 ml with tryptophan (100 μg/ml) and its control were inoculated and overnight incubated at 30 °C and 100 rpm shaking. After incubation, cultures were centrifuged at 8,000 rpm for 10 min. Two millilitres of freshly prepared Salkowski reagent (1 ml of 0.4 M FeCl_3_ in 50 ml of 35 % Perchloric acid) was added to 1 ml of culture supernatant. The reaction mixture was incubated at 30 °C for 25 min. Development of pink colour indicates the production of IAA.

### Biochemical characterisation

#### Cellulase activity

Freshly growing bacterial culture(s) were spot inoculated on nutrient agar plates supplemented with 0.2 % carboxy methyl cellulose (CMC), plates were incubated at 30 °C for 3–5 days and were overlaid with Congo-red (1 μg/ml) solution for 15 min. After washing the plate surface with 1 M NaCl, clear zone around colony indicates cellulase production.

#### Gelatin hydrolysis

Nutrient gelatin medium was inoculated with a loopful of actively growing bacterial culture and incubated for 3 days at 30 °C. Control tubes solidified when placed in ice whereas medium in inoculated tubes remained unsolidified, showing positive gelatin hydrolysis test.

#### Starch hydrolysis

Nutrient agar plates supplemented with 0.3 % soluble starch were inoculated with actively growing bacterial culture(s) aseptically and incubated for 3 days at 30 °C. When flooded with Gram’s iodine, a clear yellow zone around the inoculation spots indicates starch hydrolysis.

#### Casein hydrolysis

Skim milk agar (Skim milk powder 100 g/l, agar 15 g/l) plates were spot inoculated with actively growing culture(s) aseptically and incubated for 3 days at 30 °C. Clear zones around the inoculation spots indicate casein hydrolysis.

#### Catalase activity

Yeast extract tryptone broth tubes, inoculated with actively growing bacterial culture(s) were incubated for 3 days at 30 °C. Catalase activity was observed by adding few drops of 3 % H_2_O_2_ to the broth cultures, kept on the glass slides. Formation of oxygen bubbles confirms the positive result.

#### Denitrification test

Nitrate broth with inverted Durham tube were inoculated with actively growing culture(s), and incubated for 2–3 days at 30 °C for the observation of gas production.

#### Production of H_2_S

SIM agar [Peptone 30 g/l, Beef extract 3 g/l, ferrous ammonium sulphate 0.20 g/l, sodiumthiosulphate 0.025 g/l, agar 3 g/l (pH 6.0)] stabs were inoculated with actively growing culture(s). Blackening along the line of inoculation shows a positive test after 3–5 days at 30 °C.

### Molecular characterisation of bacterial endophytes

#### 16S rDNA amplification of genomic DNA

16S rDNA amplification from extracted DNA was performed using universal eubacterial primers, F primer 5′-AGAGTTTGATCCTGGCTCAG-3′ (*Escherichia coli* bases 8–27) and R primer 5′-TACCTTGTTTTACGACTT-3′ (*E. coli* bases 1507–1492). PCR reaction mixture (50 μl), made with 5 μl of genomic DNA, 5 μl of PCR buffer (10×), 3 μl of MgCl_2,_ 1.25 μl of dNTP mixture, 1.25 μl of forward and reverse primers each and 0.2 μl of Taq DNA polymerase. PCR reaction was performed in a thermocycler (eppendorf) and programmed as follows: Initial denaturation at 94 °C for 7 min, followed by 40 cycles of denaturation step at 94 °C for 1 min, annealing at 54 °C for 1 min, extension at 74 °C for 1 min and the final extension at 72 °C for 10 min. PCR amplicon(s) were run on 1 % agarose gel and visualised under UV gel documentation system (Gel Doc Mega-Biosystematica).

#### ARDRA-Amplified 16S rDNA restriction analysis of the amplicons

16S rDNA amplicons were digested separately with four restriction enzymes in 25 μl reaction volume containing, Amplicon 20 μl, Digestion buffer 2.5 μl (10×), Enzyme 0.1 μl and the rest being TDW (triple distilled water). Reaction mixtures containing *Msp* I, *Alu* I, *Hae* III and *Mnl* II each were incubated at 37 °C for 3 h.

#### Phylogenetic analysis

ARDRA profile of isolates was analysed on 2.5 % agarose gel. The specific band pattern was scored with binary characters (1 = presence and 0 = absence, of bands) and the phylogenetic tree was constructed using the UPGMA, a sub programme of online software http://genomes.urv.cat/UPGMA/index.php?entrada=Example2 (Garcia-Vallve et al. 1999). Cluster analysis of the isolates was done using similarity coefficient.

#### *nifH* amplification

*nifH* region of 360 bp was amplified using degenerate primer sequences (Saikia and jain 2007). Pol F primer- TGC GAY CCS AAR GCB GAC TC., Pol R primer- ATS GCC ATC ATY TCR CCG GA. PCR reaction in 50 μl reaction volume was set with: 5 μl of genomic DNA, 5 μl of PCR buffer (10×), 3 μl of MgCl_2_, 1.25 μl of dNTP mixture, 0.5 μmol of forward and reverse primers each and 0.2 μl of Taq DNA polymerase. PCR reaction was performed in a thermocycler (eppendorf) and programmed as follows: Initial denaturation at 95 °C for 5 min, followed by 35 cycles of denaturation at 94 °C for 1 min, annealing at 56 °C for 1 min, extension at 72 °C for 2 min (Chauhan 2008). PCR amplicon (s) were run on 1 % agarose gel and visualised under UV gel documentation system (Gel Doc Mega-Biosystematica).

## Results and discussion

### Isolation of bacterial endophytes from sugarcane

Based on the reported growth characteristics of *Gluconacetobacter* spp. seven suspected bacterial endophytes from different sugarcane samples were recovered, purified and assigned the respective codes (Table 1). These were observed for their shape, size, pigmentation and margin along with the colony/cell morphology of individual isolates (Table 1). Characteristic yellow/orange pigmented pellicle of *Acetobacter* appeared after 10–14 days of incubation in LGIP-agar plates; however, it takes only 5–6 days in air tight vials indicating their higher nitrogenase activity under microaerophilic environment (Cavalcante and Dobereiner 1988). All the isolates were able to grow in semi-solid LGIP medium (pH 5.5), indicating the necessity of acidic environment for these cultures (Baldani and Baldani 2005). Heavy or moderate mucous secretion was observed for all the isolates, which might help to maintain optimum O_2_ concentration without inhibiting nitrogenase activity and cell metabolism (Dong et al. 2002).

### Functional characterisation of bacterial endophytes

All isolates were found to be insensitive to the antibiotics used, except StBn and RtBn (isolates from Berinag). Both of these were inhibited significantly by Tetracyline and Metronidazole whereas Amoxyclav inhibited StBn only. This shows a significant degree of geographical sympatry among the isolates.

### Carbon source utilisation profiling

**Fig. 1:**
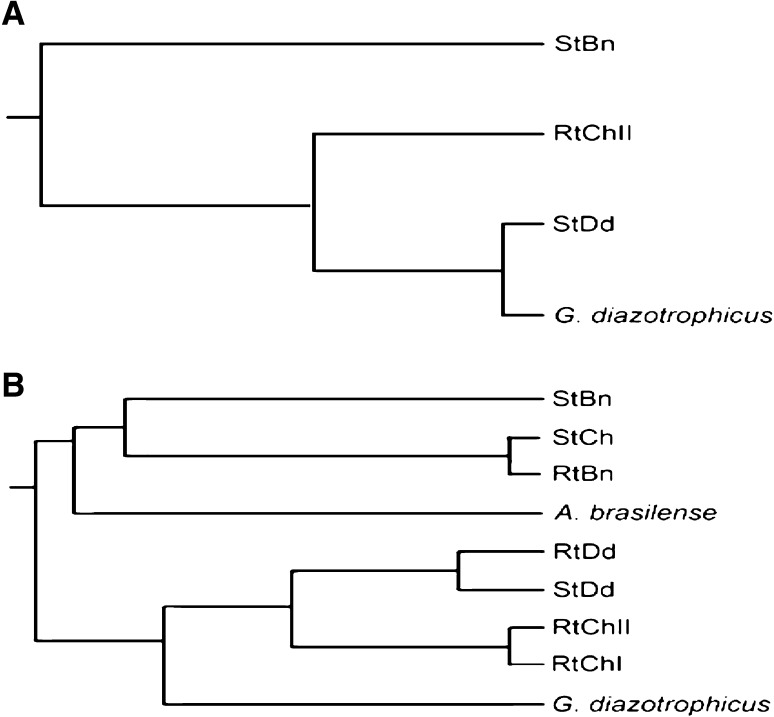
**a** Dendrogram on the basis of carbohydrate utilisation pattern of endophytic bacterial isolates. **b** Combined phylogenetic tree based on ARDRA analysis using four restriction enzymes namely *Alu* I, *Msp* I, *Hae* III and *Mnl* II

The assay produced response patterns that distinguished among widely disparate samples and among dissimilar sample types within larger categories. Differentiation of samples from different habitats was a useful preliminary test of the assay (Table 2). Carbon source utilisation profiling showed a significant relatedness among the isolates (Table 3). This involved one isolate each, StBn from Berinag (1,740 m), StDd from Didihat (1,725 m) and RtChII from Champawat (1,610 m), which were compared with the standard *G. diazotrophicus*. UPGMA hierarchical clustering showed approximately 94 % similarity between StDd and the standard strain (Fig. 1a). StDd exhibited 75 % similarity with RtChII, irrespective of its geographical closeness with StBn. Hence it can be inferred that metabolic similarity between the root isolates is more assertive than its geographical similarity.

**Table 2:** Carbohydrate utilisation pattern of endophytic bacterial isolates

Carbohydrate	StBn	RtChII	StDd	*G. diazotrophicus*
Lactose	–	+	+	–
Xylose	–	+	+	+
Maltose	–	+	–	–
Fructose	–	+	–	–
Dextrose	–	+	+	+
Galactose	–	+	+	+
Raffinose	–	+	–	–
Trehalose	–	+	–	–
Melibiose	–	+	–	–
Sucrose	–	+	–	–
L-Arabinose	–	+	+	+
Mannose	+	+	+	+
Inulin	–	–	–	–
Sodium gluconate	–	–	–	–
Glycerol	–	–	–	–
Salicin	–	–	–	–
Glucosamine	–	+	+	+
Dulcitol	–	+	+	+
Inositol	–	+	+	+
Sorbitol	–	–	–	–
Mannitol	–	+	–	–
Adonitol	–	+	–	–
α-Methyl-D-glucoside	–	–	–	–
Ribose	–	–	+	+
Rhamnose	–	+	–	–
Cellobiose	–	–	–	–
Melezitose	–	–	–	–
α-Methyl-D-mannoside	–	–	–	–
Xylitol	+	+	+	+
ONPG (O-nitrophenyl-β-D-galactopyranoside)	–	+	+	+
Esculin	–	+	+	+
D-Arabinose	–	+	+	+
Citrate	–	+	+	+
Malonate	–	+	+	+
Sorbose	–	–	–	–

(Positive test ^+^, Negative test ^−^)

**Table 3:** Similarity matrix for the endophytic bacterial isolates and their Jaccard coefficient values based upon the dendrogram generated for the carbon source utilisation profiling for the selected strains and the standard used

Similarity matrix computed with Jaccard coefficient				
	StBn	RtChII	StDd	*G. diazotrophicus*
StBn	1	0.04	0.05	0.05
RtChII		1	0.60	0.56
StDd			1	0.93
*G. diazotrophicus*				1

### Plant growth promotory studies

#### Phosphorous solubilisation

Except StBn, all the isolates showed clear zones on Pikovaskya medium (Pedraza 2008), indicating positive test for P-solubilisation (Table 1). In spite of the fact that soil usually contains high amounts of total phosphorous, its availability to plants is always scarce and thus a limiting factor.

#### Siderophore production

All the isolates produced siderophore on CAS-agar except RtBn (Table 1). Bacterial plant growth promotion (PGP) can result from direct or indirect mechanisms, including siderophores production. They can improve vegetative growth by increasing plant nutrient availability through iron uptake and preventing the growth of soil borne pathogens due to iron limitation (Chaiharn et al. 2009; Miethke and Marahiel 2007).

#### Indole acetic acid (IAA) production

Out of seven isolates used in this study, only two isolates (StBn and RtBn) showed IAA production in the range of 7.2–8.9 μg/ml in the presence of tryptophan in YPM (Table 1). The YPM medium supplemented with tryptophan acts as precursor of IAA. PGP is accomplished by different mechanisms, such as, solubilisation of essential minerals, increased nutrient uptake, production of certain phytohormones like IAA, vitamins, enzymes and suppression of pathogens through siderophores or by bio-control agents.

#### Biochemical characterisation

All the isolates were H_2_S producing and none of the isolates showed any response towards gelatin hydrolysis, starch hydrolysis and cellulase production (Table 1). Except RtChII, none of the isolates showed casein hydrolysis. Catalase activity was shown by all the isolates except RtChI and RtDd. The denitrification test was positive in case of RtDd, RtChI and RtChII. Hence these properties can be ascertained as an index of similarity or relatedness among the isolates and also signifies their agricultural importance.

### Molecular characterisation of the isolates using ARDRA

**Fig. 2:**
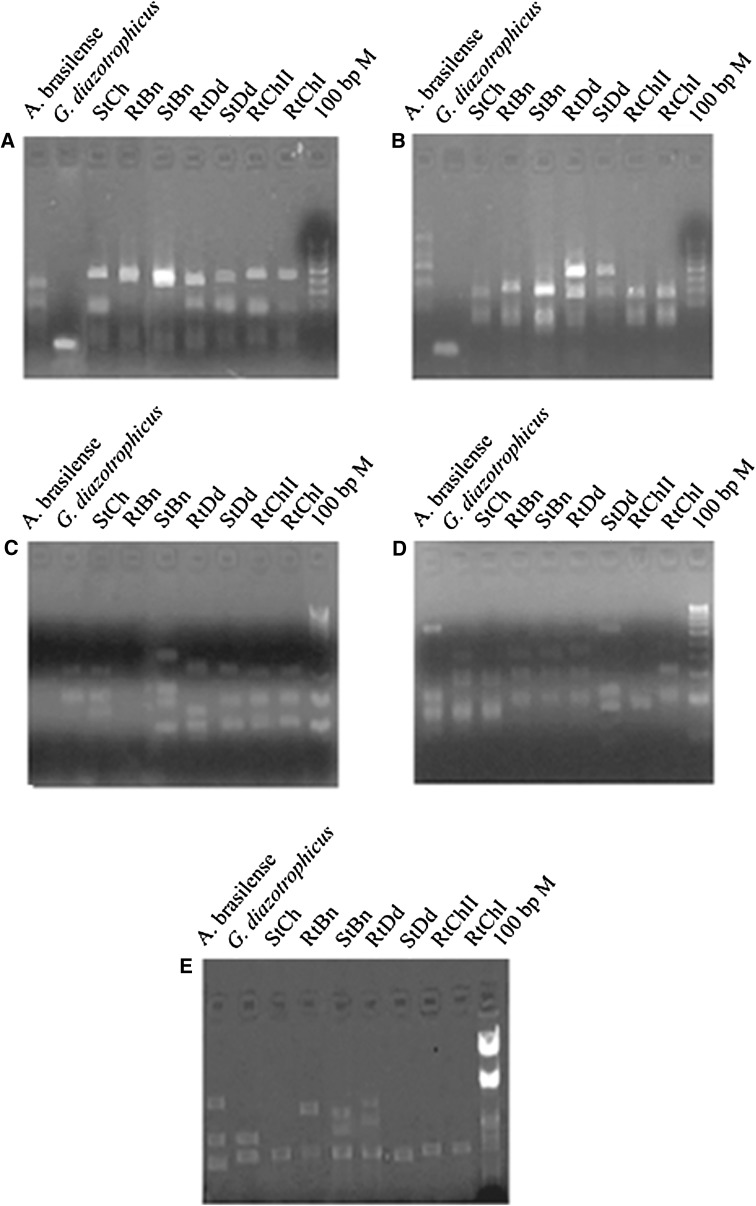
Restriction digestion of the 16S rDNA amplicon, using enzymes. **a***Alu* I, **b***Msp* I, **c***Mnl* II, **d***Hae* III, **e***nifH* amplification of the isolates and standards

ARDRA and subsequent UPGMA analysis offers a powerful tool for bacterial species identification. 16S rDNA amplicons of the isolates and standards were restriction digested individually with *Alu* I, *Msp* I, *Hae* III and *Mnl* II (Fig. 2a–d). For any isolate, to belong to a given species, there must be at least 80–85 % similarity based upon unweighted pair group method analysis. UPGMA cluster analysis resulted in two major clusters based upon the similarity index calculated using Jaccard coefficient (Table 4). Cluster I contained StBn with 40 % similarity to StCh and RtBn, whereas all these isolates showed significantly lesser (<40 %) similarity with *A. brasilense*. Cluster II included RtDd and StDd which were nearly 90 % similar. Isolates RtChII and RtChI were identical with 100 % similarity. Together these isolates were assigned the similarity in the range of 30–50 % to another standard used in the study, *G. diazotrophicus* (Fig. 1b). A 1,400 bp amplicon from all the isolates confirmed their eubacterial nature. Together these isolates were less than 55 % similar to both *A. brasilense* and *G. diazotrophicus*, which can be attributed to their different geographical pertaining. However, they showed significant homology varying from 55 to 70 % as per their geographical kinship in terms of altitudes from sea level.

**Table 4:** Similarity matrix for the endophytic bacterial isolates and their Jaccard coefficient values based upon the dendrogram generated for the combined phylogenetic (ARDRA analysis) using four restriction enzymes namely *Alu* I, *Msp* I, *Hae* III and *Mnl* II

Similarity matrix computed with Jaccard coefficient									
	St.Bn.	Rt.Dd.	St.Dd.	Rt.Ch.II.	Rt.Ch.I	St.Ch.	Rt.Bn.	*A. brasilense*	*G. diazotrophicus*
St.Bn.	1	0.50	0.53	0.40	0.40	0.40	0.40	0.36	0.33
Rt.Dd.		1	0.90	0.69	0.69	0.25	0.25	0.45	0.41
St.Dd.			1	0.61	0.61	0.27	0.27	0.50	0.33
Rt.Ch.II.				1	1	0.15	0.15	0.33	0.54
Rt.Ch.I					1	0.15	0.15	0.33	0.54
St.Ch.						1	1	0.28	0.11
Rt.Bn.							1	0.28	0.11
*A. brasilense*								1	0.10
*G. diazotrophicus*									1

### *nifH* amplification

*nifH* amplification was observed in all the recovered isolates, *Azospirillum* and *Gluconacetobacter* isolates with a common band of nearly 400 bp (Fig. 2e). Multiple bands were observed in StBn, RtBn, StDd and standards. This may be due to the use of degenerate primers which amplify some of the non specific regions along with *nifH* gene (Ando et al. 2005). *nifH*, being the oldest existing functional genes in evolutionary history, was used for evaluating the relatedness among the isolates supplementary to ARDRA analysis. *nifH* PCR amplicons reflect genetic potential and thus also reflect genetic potential for nitrogen fixation in a particular environment. PCR using degenerate primers enables diverse group of microorganisms to be detected. Moreover, it also signifies the agricultural relevance of the isolates as potential nitrogen fixers.
